# Fatty Acid Transporter CD36 Mediates Hypothalamic Effect of Fatty Acids on Food Intake in Rats

**DOI:** 10.1371/journal.pone.0074021

**Published:** 2013-09-06

**Authors:** Valentine S. Moullé, Christelle Le Foll, Erwann Philippe, Nadim Kassis, Claude Rouch, Nicolas Marsollier, Linh-Chi Bui, Christophe Guissard, Julien Dairou, Anne Lorsignol, Luc Pénicaud, Barry E. Levin, Céline Cruciani-Guglielmacci, Christophe Magnan

**Affiliations:** 1 Unit of Functional and Adaptive Biology, EAC 4413 CNRS, Université Paris Diderot, Paris, France; 2 Department of Neurology and Neurosciences, NJ Medical School, Newark, New Jersey, United States of America; 3 IFR150 STROMA Lab (UMR 5273), Université Paul Sabatier – CNRS-EFS-INSERM U1031, Toulouse, France; 4 Centre des Sciences du Goût et de l’Alimentation, UMR 6265 CNRS, 1324 INRA, Université de Bourgogne, Dijon, France; 5 Neurology Service, VA Medical Center, East Orange, New Jersey, United States of America; INSERM/UMR 1048, France

## Abstract

Variations in plasma fatty acid (FA) concentrations are detected by FA sensing neurons in specific brain areas such as the hypothalamus. These neurons play a physiological role in the control of food intake and the regulation of hepatic glucose production. Le Foll et al. previously showed *in vitro* that at least 50% of the FA sensing in ventromedial hypothalamic (VMH) neurons is attributable to the interaction of long chain FA with FA translocase/CD36 (CD36). The present work assessed whether *in vivo* effects of hypothalamic FA sensing might be partly mediated by CD36 or intracellular events such as acylCoA synthesis or β-oxidation. To that end, a catheter was implanted in the carotid artery toward the brain in male Wistar rats. After 1 wk recovery, animals were food-deprived for 5 h, then 10 min infusions of triglyceride emulsion, Intralipid +/− heparin (IL, IL_H_, respectively) or saline/heparin (S_H_) were carried out and food intake was assessed over the next 5 h. Experimental groups included: 1) Rats previously injected in ventromedian nucleus (VMN) with shRNA against CD36 or scrambled RNA; 2) Etomoxir (CPT1 inhibitor) or saline co-infused with IL_H_/S_H_; and 3) Triacsin C (acylCoA synthase inhibitor) or saline co-infused with IL_H_/S_H_. IL_H_ significantly lowered food intake during refeeding compared to S_H_ (p<0.001). Five hours after refeeding, etomoxir did not affect this inhibitory effect of IL_H_ on food intake while VMN CD36 depletion totally prevented it. Triacsin C also prevented IL_H_ effects on food intake. In conclusion, the effect of FA to inhibit food intake is dependent on VMN CD36 and acylCoA synthesis but does not required FA oxidation.

## Introduction

The central nervous system (CNS) is a key player in the regulation of energy balance in mammals [1], [2]. This process involves a combination of signals arising from the periphery, including hormones (leptin, insulin, ghrelin etc.) and nutrients (glucose and fatty acids, FA), which are detected within brain areas such as the hypothalamus and brainstem [3], [4], [5], [6]. Since the work of Oomura et al [7] several lines of evidence support the idea that specialized hypothalamic metabolic sensing neurons can monitor peripheral fuel availability by altering their activity in response to ambient levels of FA as a means of regulating energy and glucose homeostasis in the body [1], [3], [5], [8]. Regulation of energy balance through such hypothalamic FA sensing includes insulin secretion and action, hepatic glucose production and food intake [8], [9], [10], [11]. Molecular mechanisms relaying the effect of FA are still a matter of debate. Prolonged intracerebroventricular infusion of oleic acid (OA) decreases both food intake and glucose production in rats through a K_ATP_ channel dependent mechanism [11]. Both mitochondrial reactive oxygen species [12] and nitric oxide production [13] have been also evidenced as mediators for brain lipid sensing in rats. Many of these effects may be mediated by hypothalamic FA sensing neurons. Le Foll *et al.* [14] previously showed *in vitro* that at least 50% of the FA sensing in VMH neurons (arcuate nucleus+ventromedian nucleus) is attributable to the interaction of long chain FA with FA translocase/CD36 (CD36) while only ∼20% is attributable to intracellular metabolism of FA. The present work was aimed at studying the potential role of neuronal FA sensing, as mediated by CD36 and/or intracellular FA metabolism, in the regulation of refeeding. To that end 5 h fasted rats were infused for 10 min with a heparinized triglyceride emulsion (Intralipid, IL_H_) through carotid artery and spontaneous food intake was monitored over the next 5 h of refeeding. Such short term carotid infusion of IL_H_ was designed to mimic the increase in TG-enriched lipoproteins to which the brain is exposed post-prandially. These studies were also designed to assess whether LPL-dependent hydrolysis might occur to locally increase FA availability as recently evidenced by Wang *et al* [15], [16]. We found that acute IL_H_ infusions decreased spontaneous food intake during refeeding independently of β-oxidation but through mechanisms involving both CD36 and acylCoA synthesis.

## Materials and Methods

### Ethics Statement

The experimental protocol was approved by the institutional Animal Care and Use Ethical Committee of the Paris-Diderot University (registration number CEEA-40).

### Animal Models

Two-month-old male Wistar and Sprague Dawley rats (225–250 g, Charles River, l’Arbresle, France) were used. They were housed individually in stainless steel cages in a room maintained at 22±1°C with lights on from 0700 am to 0700 pm. They were given a standard laboratory diet (19.4%; protein, 59.5%; carbohydrate 4.6% fat of total energy content, 16.5% vitamins and minerals and water *ad libitum*).

### Materials

Intralipid is a triglyceride emulsion composed with saturated fatty acids (16%) and unsaturated fatty acids (23% mono- and 61% poly-unsaturated) (Fresenius Kabi, Aubervilliers, France). For some experiments, heparin (Sanofi-Aventis, Reuil-Malmaison, France) was added to the Intralipid infusates to stimulate triglyceride hydrolysis [17]. Both etomoxir,to inhibit CPT1 activity, and triacsin C, to inhibit acylCoA synthase activity, were provided by Sigma-Aldrich (Vitry-sur-Seine, France).

### Short Term Infusion Toward the Brain

Short-term intracarotid infusions were performed to assess the effects of FAs on forebrain FA sensing independently of their variations in blood stream. To that end, animals were anesthetized with isoflurane (1.5% at 0.8 l/min) and xylazine (Rompun, 10 mg/kg) and a catheter was inserted in the left carotid artery facing toward the brain, as previously described [18]. Ten days after recovery, animals were food-deprived for 5 h. Then 10 min infusions were carried out and food intake was assessed over the next 5 h. This protocol was repeated daily for 3d in each animal. During the first 2d, animals were infused with saline at 20 µl/min. The third day, they received Intralipid (IL) or heparinized-Intralipid (IL_H_) at 20 µl/min (IL). Controls were infused with saline (S) or heparinized-saline (S_H_) at the same rate of infusion. To test the role of acylCoA synthesis or β oxidation, triacsin c and etomoxir were co-administered with IL_H_ or S_H,_respectively, in two different series of experiments. Etomoxir and triacsin C were prepared in saline and added to infusion solutions at 150 µM and 80 µM, respectively [9], [19]. A third set of studies was designed to assess the role of VMNCD36 as a mediator of feeding. These animals were bilaterally injected with scrambled- vs shRNA anti-CD36 into VMN (see below) after insertion of catheter into carotid artery 10d prior to the intracarotid infusion studies. At the end of the experiment, hypothalami were collected and stored at −80°C until measurement of CD36 mRNA expression.

### Design and Injection of FAT/CD36 shRNA

ShRNA was obtained from Cliniscience (Montrouge, France). The sequence was TTGTACCTATACTGTGGCTAAATGAGAC. ShRNA was expressed in a pGFP-V-RS retroviral vector. The shRNA efficiency was tested with *in vitro* co-transfection of HEK293 cells with a PsyCheck vector containing the CD36 sequence coupled with a luciferase. The assessment of shRNA efficacy was done by measuring of luminescence made by luciferase (dual-glo luciferase assay, Promega, Valence, France). The system of transfection was non-viral (JetPrime, Polyplus-transfection, Pantin, France).

### Anti-CD36 shRNA Administration in VMN

Anti-CD36 shRNA was prepared in jetPEI solution (JetPEI, Polyplus-transfection, France). Briefly, 10 µg of DNA was mixed with JetPEI in order to obtain an N/P ratio of 8. The mix was dissolved in glucose solution at a final concentration of 5%. Three µl of this mixture containing 1.5 µgof scrambled or CD36 shRNA were infused bilaterally into VMN of rats10d prior to the intracarotid infusion studies.The rate of infusion was 0.15 µl/min during 20 min to avoid brain lesions. Coordinates were medio-lateral +/−0.6 mm and anteroposterior −2.4 mm compared to bregma and −9.6 mm deep to the top of the brain [20].

### Microdialysis

VMH microdialysis was performed in a separate group of rats to measure the FA content in response to intracarotid IL, IL_H_ and S_H_ infusions (n = 7/group). One week before the study, animals were anesthetized with chloropent (0.3 ml/100 g body wt, ip;pentobarbital, chloral hydrate, and magnesium sulfate). A catheter was inserted in the left jugular vein and a catheter was inserted in the left carotid artery facing toward the brain. The day before the infusions, rats were anesthetized with isoflurane (1.5% at 0.8 l/min) and were implanted stereotaxically with a FA microdialysis probe (MAB 5.15.3PE, Microbiotech/se AB, Stockholm, Sweden) in the VMH. Coordinates were 3.3 mm medial-lateral, 3.4 mm anteroposterior to Bregma and 9.2 mm deep to the top of the brain [20]. The probes were implanted at an angle of 20° and fixed with dental cement to the skull. The day of the experiment, animals were connected to lines filled with artificial cerebrospinal fluid (Harvard Apparatus, Holliston, Massachusetts, USA) containing 3% fatty acid free bovine serum albumin (Sigma Aldrich, St. Louis, Missouri, USA) and perfused at a rate of 1.0 µl/min. Microdialysate eluates and blood samples were collected every 30 min during food deprivation and over the 5 h following the 10 min infusions. Plasma was collected and samples were stored at −80°C until NEFA assay. Animals were killed at the end of this experiment to assess probe placement.

### C-fos Immunochemistry

Rats were anaesthetised with pentobarbital 1 h after the lipid infusion and transcardially infused with ice-cold 0.9% saline for 10 min followed by a 20 min infusion of 4% paraformaldehyde in PBS. Brains were removed and post-fixed in ice-cold 4% paraformaldehyde for 2 h, after which they were cryoprotected in 30% sucrose in PBS for 2 to 3 days at 4°C. They were then frozen and cut into 40 µm coronal sections on a freezing cryostat. Free-floating sections were rinsed in PBS and exposed to 0.3% hydrogen peroxide for 30 min. They were then preincubated in PBS containing 3% normal goat serum and 0.25% Triton (blocking solution) for 2 h and incubated overnight at 4°C with rabbit polyclonal anti-c-fos serum (1∶10,000 dilution; Ab-5; Oncogene Sciences, San Diego, CA, USA) in blocking solution. Subsequently, sections were incubated with biotinylated goat anti-rabbit IgG diluted at 1∶1000 (Jackson Laboratories, Burlingame, CA, USA) for 1 h and with streptavidin horseradish peroxidase for 1 h, both in blocking solution. C-fos expression was visualised for fos-like immunoreactivity (FLI) using diaminobenzidine and hydrogen peroxide in distilled water. Several PBS rinses were carried out between the above steps, except between blocking and incubation with primary antibody. Sections were mounted on silanized slides, dehydrated in alcohol, cleared in Bioclear (MicroStain (D-limonene), Micron, Francheville, France) and examined under a transmitted-light microscope (DMRB Leica, Gennevilliers, France).

### Counting the c-fos-like Immunoreactive Neurons

Fos-immunoreactive cells without distinction of labeling intensity were counted bilaterally in different cerebral regions by using a computerized image analysis (Image J). Between 6 and 12 sections per region were analyzed. Results were expressed as the mean of the sum of Fos-positive nuclei counted per pixel^2^ in each region of interest. This quantification was made for the paraventricular (PVN), arcuate(ARC) and the ventromedian nuclei (VMN). The cerebral cortex (CC) was chosen as a non-lipid sensitive area.

### Measurement of Hypothalamic FAconcentrations by GC-MS

Hypothalamus and cortex were weighed, homogenized in methanol. 50 µL of homogenate were mixed with BF_3_ (14%)/methanol and 10 µg of heptadecanoic acid as an internal standard were added. Samples were heated (100°C; 40 min) then cooled at room temperature. Heptane/distilled water (1∶2) was added, samples were vortexed for 30 sec then centrifuged for 2 min at 3000 rpm. The supernatant was collected and evaporated with a Speedvac (Jouan, Saint-Herblain, France). Dry samples were solubilized in heptane. OneµL FA methyl esters (FAMEs) was analyzed on GC-MS instrument: with 1/100 split wherein a Shimadzu was interfaced with a GC2010 mass selective detector. The mass spectra and retention indices registered in the FAMEs GC/MS library were obtained using the Shimadzu GCMS-QP2010. This was done using the SLB-5 ms and the Supelcowax-10 columns (length 30 m×inner diameter 0.25 mm×film thickness 0.25 µm) made by Sigma-Aldrich Supelco (Lizy sur Ourcq, France).

### Plasma Assays

Enzyme assay kits were used to determine both plasma FAs (NEFA-C test, Wako, Frankfurt,Germany) and triglycerides concentrations (Boehringer-Mannheim, Ulm, Germany).

### RNA Preparation and Real-time qPCR Analysis

Animals were sacrificed 5 h post-infusion and either VMH (POMC, CART, NPY, AgRP) or hypothalamus (GPR119, GPR120, FATP1, CD36) was removed. Total RNAs were isolated using RNeasy Lipid mini kit (Qiagen, Aubervilliers, France). To remove residual DNA contamination, the RNA samples were treated with DNAseRNAse-free (Qiagen, Aubervilliers, France). Four µg of total RNA from each sample was reverse transcribed with 40 U of M-MLV Reverse Transcriptase (Invitrogen, life technologies, Logan, UT, USA) using random hexamer primers. The primer sequences were as follows: CD36 S GCCTCCTTTCCACCTTTTGT, CD36 AS GATTCAAACACAGCATAGATGGAC,FATP1 S GGGTTTGCAAGCCAGAGA, FATP1 AS CAAAGCAGCCCCAATGAG, GPR119 S CCTATTGGCAGAGGGAGGT, GPR119 AS CTGCCATCAGCAAGTAGCC, GPR120 S TGATCAGCTACTCCAAGATTTTACA, GPR120 AS GAAGAGCGTTCGGAAGAGC,NPY S GCCCGCCATGATGCTAGGTAA, NPY AS GGGGTACCCCTCAGCCAGAA, POMC S CCAGGACCTCACCACGGAA, POMC AS GACGTACTTCCGGGGATTTTCA, AgRP S CGAGTCCTGCTTGGGACAACA, AgRP AS GCAGAGGTTCGTGGTGCCAGTA, CART S GCGCTGTGTTGCAGATTGAA, CART AS CCCCTTCACAAGCACTTCAAGA. Housekeeping gene was rpl19 S GCTGAGGCTCGCAGGTCTAA, rpL19 AS CAGACACGAGGGACGCTTCA. Real time quantitative PCR amplification reaction was carried out in a LightCycler 480 detection system (Roche, Parthenay, France) using the LightCycler 480 SYBR Green I Master (Roche, Parthenay, France). 40 ng of reverse transcribed RNA was used as template for each reaction. All reactions were carried out in duplicate with no template control. The PCR conditions were: 95°C for 5 min, followed by 40 cycles of 95°C for 10 sec, 60°C for 10 sec and 72°C for 10 sec. The mRNA transcript level was normalized against rpL19. To compare mRNA level, relative quantification was performed as outlined in Pfaffl *et al* [21]. Ratio = (Eff target)^ΔCptarget(MEANcontrol – MEANsample)^/(Eff ref.)^ΔCpref(MEANcontrol-MEANsample).^

### Statistical Analysis

Data are means ± sem. Statistical analysis was performed using Graphpad Prism software (Draveil, France). Comparisons of groups were made using a non-paired Student’s t test or a one way repeated measures ANOVA with post hoc Bonferroni test. Differences among groups were considered significant when p<0.05.

## Results

### Effects of IL and IL_H_ Carotid Infusion on Food Intake

**Figure 1:**
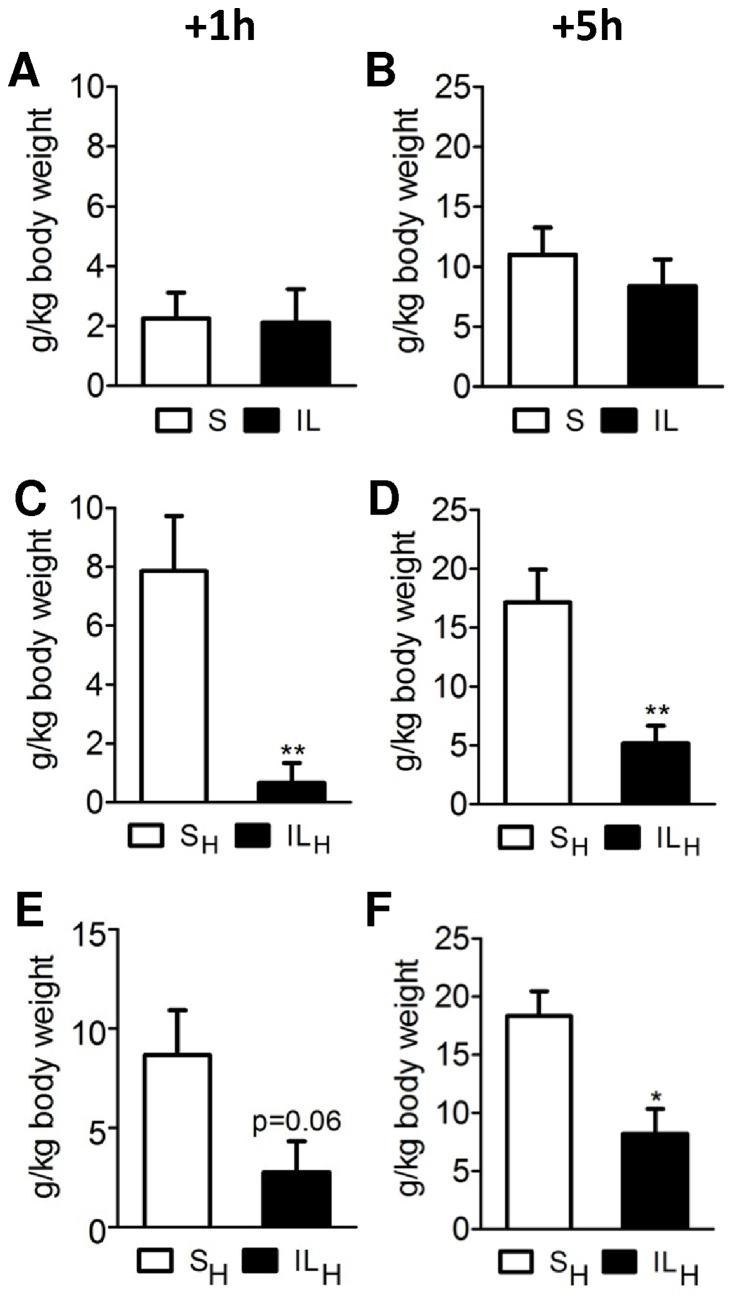
Food intake measurement after 10 min infusion toward brain of saline (S; open bars; control) and Intralipid 20% at 20 µL/min (IL; solid bars) without (A, B) or with heparin (C,D) in Wistar rats. The same experiment was realized in Sprague Dawley rats (E,F). A, C, E: 1 h-food intake. B, D, F: 5 h-food intake. Values are means ±SEM; n≥6 rats/group. *p<0.05, **p<0.01, significantly different from controls.

Intracarotid infusions of intralipid alone did not induce changes in food intake compared to saline infusions during 1 h and 5 h of refeeding (fig. 1A and 1B). In contrast, when heparin was added to intralipid (IL_H_) food intake was significantly reduced compared to S_H_ infusions at 1 h (−91%; 1C) and 5 h (−70%; 1D) after the 10 min infusions in Wistar rats. Similar results were obtained with Sprague Dawley rats (1E and 1F) with a significant 55% decrease of food intake 5 h after infusion. However, there was no effect of IL_H_ infusions over the next 24 h (data not shown). These results suggest that forebrain FAs were involved in the control of food intake. As there was no effect of either saline or intralipid alone, further experiments were only performed in S_H_ vs IL_H_ rats. Note that both plasma FA (403.4±47.3 µM in S_H_ vs 404.5±53.5 µM in IL_H_ rats) and TG concentrations (0.8±0.3 mg/ml in S_H_ rats vs 0.7±0.2 mg/ml remained similar in S_H_ vs IL_H_ during the experimental period. Consistently with the reduced food intake, glycemia was lower in ILH rats compared to controls at 1 h and 5 h after the 10 min infusions (108±3.5 mg/dl for SH vs 92.8±3.2 m/dl for ILH; p<0.01).

### Measurement of VMH Free FA Levels

**Figure 2:**
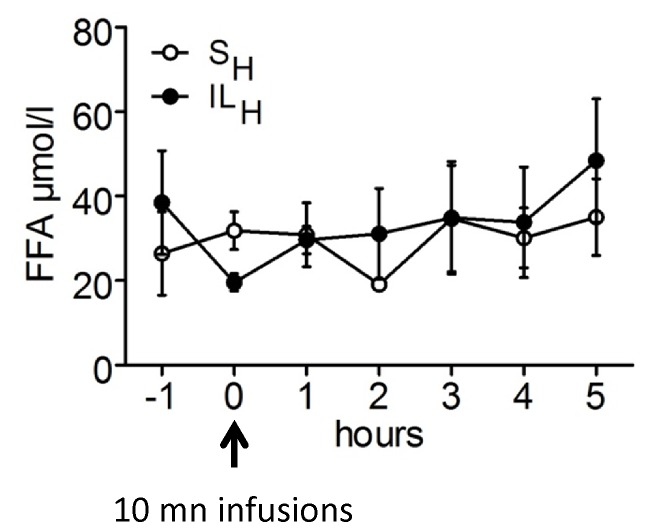
Microdialysis fatty acids concentrations before and after 10 min infusion toward brain of S_H_ or IL_H_. A: area under the curve (AUC) for basal (all rats before infusions), S_H_ and IL_H_ groups. AUC is calculated 30 min before the 10 min infusion and 1 h after. B: Time course of microdialysis fatty acids with 0 which is the end of the infusion. Values are means ±SEM; n≥6 rats/group. *p<0.05 significantly different from basal.

To investigate the effect of IL_H_ infusion on the FAs concentration in VMH, we did microdialysis experiment in a series of Sprague Dawley rats. Figure 2 displayed time course of FA concentration in VMH of S_H_ vs IL_H_ following infusions. There was no difference in both groups by repeated measures analysis of variance. In addition, analysis of FA concentrations in whole hypothalamus by GS-MS also showed no difference between S_H_ and IL_H_ groups with the exception of a decrease in vaccenic acid in hypothalamus of IL_H_ rats (Table 1).

**Table 1:** Composition in FA in whole hypothalamus measured by GS-MS and expressed in percentage.

		Hypothalamus		Cortex	
		S_H_	IL_H_	S_H_	IL_H_
**Saturated FAs**	Myristic acid 14∶0	0.029±0.002	0.027±0.002	0.038±0.002	0.035±0.002
	Palmitic acid 16∶0	30.53±0.32	30.60±0.31	36.40±0.5	36.71±0.47
	Stearic acid 18∶0	29.65±0.36	29.94±0.32	34.00±0.4	34.13±0.29
**MUFAs**	Oleic acid 18∶1 ω9	22.01±0.22	22.11±0.19	13.85±0.4	13.45±0.34
	Vaccenic acid 18∶1 ω7	6.75±0.15	6.16±0.20*	4.10±0.2	3.98±0.15
**PUFAs**	Linoleic acid 18∶2 ω6	0.032±0.002	0.034±0.004	0.032±0.004	0.028±0.001
	Arachidonic acid 20∶4 ω6	5.72±0.16	6.01±0.16	6.33±0.2	6.39±0.20
	Docohexanoic acid 22∶6 ω3	5.27±0.21	5.13±0.19	5.24±0.2	5.27±0.17
**%**	Saturated FAs	60.2±0.5	60.6±0.4	70.4±0.4	70.9±0.5
	MUFAs	28.8±0.3	28.3±0.2	18.0±0.4	17.4±0.5
	PUFAs	11.0±0.3	11.2±0.3	11.6±0.4	11.7±0.2
**ratio**	MUFAs/PUFAs	2.62±0.08	2.55±0.08	1.56±0.08	1.50±0.05
	MUFAs/Saturated FAs	0.48±0.01	0.47±0.01	0.26±0.01	0.25±0.01
	PUFAs/Saturated FAs	0.18±0.01	0.18±0.01	0.16±0.01	0.17±0.00

* p<0.05 vs. S_H_ group.

### Hypothalamic c-fos and Gene Expression Following Intracarotid Infusions

**Figure 3:**
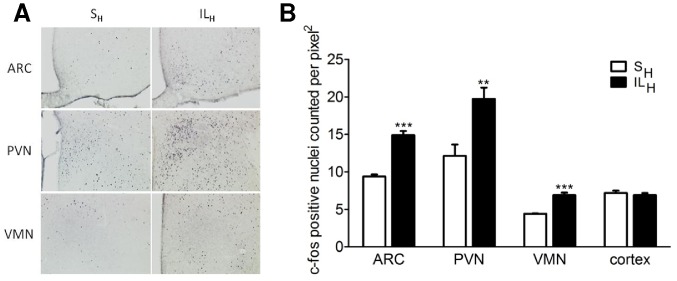
Immunostaining of c-fos positive cells in hypothalamic nuclei (A, B) and neuropeptides expression (C) in hypothalamus after 10 min infusion toward brain of S_H_ or IL_H_. A: photomicrographs showing c-fos positive cells localization in arcuate nucleus (ARC), paraventricular nucleus (PVN) and ventromedian nucleus (VMN) in controls (left) and IL_H_ rats (right). B: number of c-fos-positive nuclei counted per pixel2 in controls (open bars) and ILH rats (solid bars). Values are means ±SEM; n≥6 rats/group. **p<0.01, ***p<0.001 significantly different from controls.

To assess which hypothalamic areas were involved in the FA effect on food intake, we assessed c-fos immunostaining in the hypothalamus at 1 h after intracarotid infusions. The number of c-fos positive cells was significantly increased by about 60% in ARC, PVN and VMN of IL_H_ vs S_H_ (figure 3A and 3B). There was no difference in hypothalamic NPY/AgRP and POMC/CART gene expression between both groups (data not shown).

### Gene Expression of CD36, FATP1, GRP119 and GRP120 in Hypothalamus

**Figure 4:**
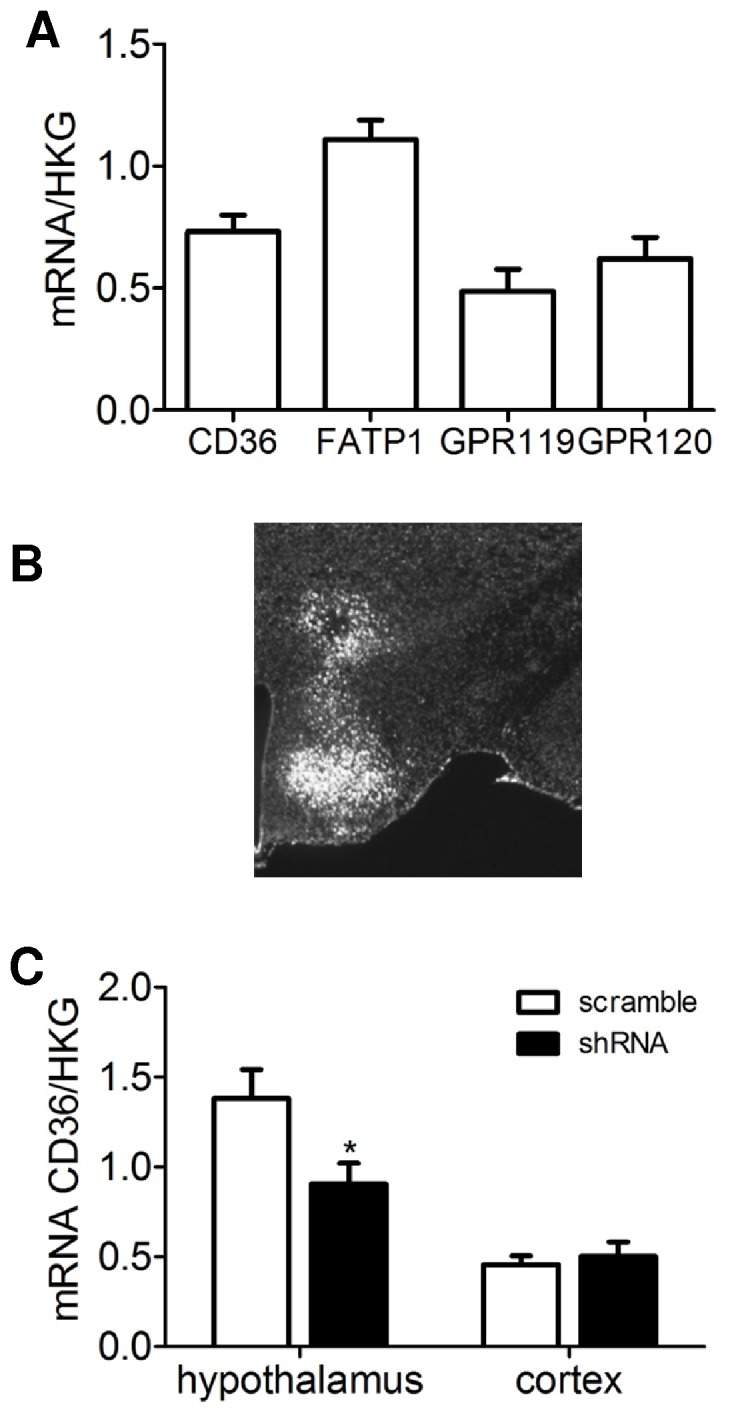
Hypothalamic mRNA expression normalized with HKG. A: expression of different fatty acids transporters. B: photomicrograph showing the JetPEI injection site. C: expression of CD36 in shRNA and scramble rats. Values are means ±SEM; n≥5 rats/group. *p<0.05, significantly different from scramble rats.

All four genes were detected and expressed to the same level in hypothalami of rats (figure 4A). To decrease CD36 expression in VMN, shRNA was specifically infused as shown on the figure 4B. Injection of shRNA against CD36 induced a decrease of CD36 gene expression by about 35% compared to scramble (figure 4B).

### Food Intake Measurements

**Figure 5:**
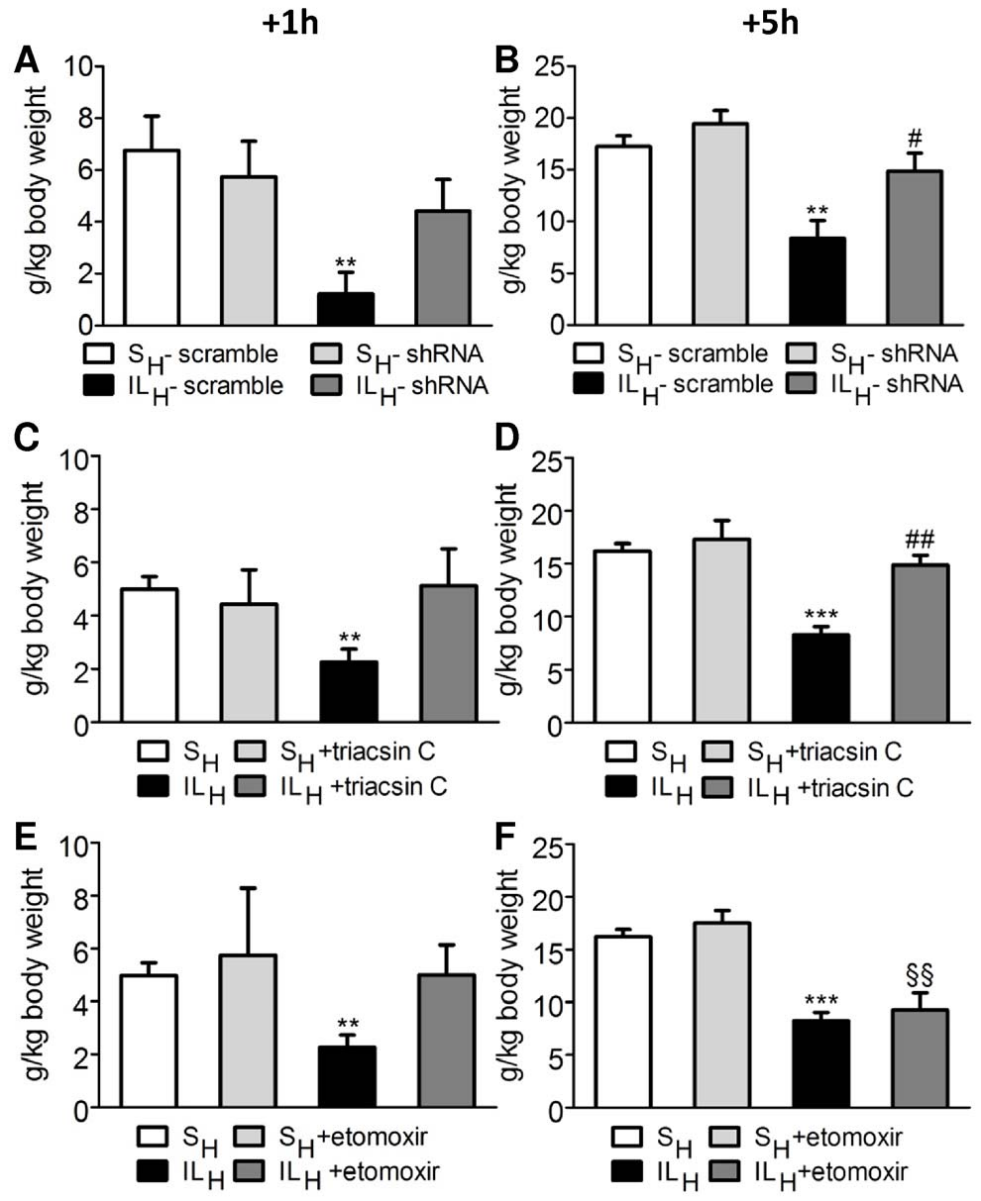
Food intake measurement after 10 min infusion toward brain of S_H_ (open bars; control), S_H_+treatment (light grey bars), IL_H_ (solid bars) and IL_H_+treatment (dark grey bars) groups. A, B: 1 h-food intake (A) and 5 h-food intake (B) in scramble and CD36 shRNA rats infused with S_H_ or IL_H_. C, D: 1 h-food intake (C) and 5 h-food intake (D) in S_H_ and IL_H_ groups co-infused with triacsin C (80 µM). E,F: 1 h-food intake (E) and 5 h-food intake (F) in S_H_ and IL_H_ groups co-infused with etomoxir (150 µM). Values are means ±SEM; n ≥4 rats/group. ***p<0.001, significantly different from S_H_; ^§§^p<0.01 significantly different from S_H_+treatment. ^#^p<0.05, ^##^p<0.01 significantly different from IL_H_.

To evaluate the potential mechanisms by which IL_H_ infusion reduced food intake, rats had VMN CD36 expression reduced using CD36 shRNA or were infused with either S_H_ or IL_H_ containing etomoxir or triacsin C. VMN CD36 shRNA injections reduced hypothalamic CD36 gene expression by 34% (figure 4B). In these rats, the inhibitory effect of IL_H_ infusion on food intake compared to S_H_ was lost (figure 5A and 5B). Whereas 5 h intake was reduced by ∼70% in IL_H_ vs. S_H_ rats, there was no such IL_H_-associated decrease in rats with VMNanti-CD36 shRNA injections. Similarly, inhibition of acylCoA synthase with triacsin C, the IL_H_-induced reduction in food intake at 5 h, as well as 1 h, was blocked (figure 5C and D). In contrast, this inhibitory effect of IL_H_ persisted at 5h after refeeding when CPT1 was inhibited by co-administration of etomoxir with IL_H_ (figure 5F). After 1 h (figure 5E), there was a reversal of intralipid-induced feeding inhibition, but this effect was transient.

## Discussion

Since the work by Oomura *et al* [7], growing evidence suggests that neurons can sense FA and that hypothalamic FA sensing plays a role in the regulation of food intake [3], [5]. Also dysfunction of this FA sensing may contribute to the further deterioration of the energy balance and finally to obesity, with or without type 2 diabetes as a complicating factor [8], [22]. Despite the fact that intracerebroventricular infusion of oleic acid has been shown to decrease both food intake and hepatic glucose production [11], the idea that an increase in brain FA levels in response to a meal could act as a satiety signal to inhibit feeding appears counterintuitive. Indeed, plasma FA levels do decrease significantly after food ingestion [23]. On the other hand, levels do rise significantly during fasting [24] a situation in which food intake would be expected to increase. This issue was addressed by Oomura *et al* in the abstract of their previously cited work *“It may be suggested that when the rat is in energy deficit and FFA is consequently released into the blood stream, the specific chemosensitive neuron might sense this event and lead the animal to eat.”* [7]. In the same vein, the feeding response is experimentally activated by systemically administered drugs such as β-mercaptoacetate [25] or etomoxir [26] that reduce FA oxidation. Thus, if plasma FAs are not responsible for decreased food intake during a meal, other mechanisms may be involved to locally provide FA in hypothalamus in post-prandial state. Plasma levels of triglyceride (TG)-enriched lipoproteins do rise after food ingestion [23], [27] and could represent physiologically relevant signals to modulate energy balance including food intake. Indeed, post-prandial TG-enriched particles are abundant lipid species which are hydrolyzed by the lipoprotein lipase (LPL) and recent studies have highlighted a role for neuronal LPL-mediated hydrolysis of TG particles in the regulation of energy balance [15], [16].

In the present study we designed an experimental procedure aimed at mimicking an increased concentration of TG-enriched particles which could reach the brain in post-prandial state and locally provide substrate to LPL, thus leading to production of FAs. FAs may in turn act as signal targeting hypothalamic FA sensing neurons resulting in decreased food intake. To that end rats were infused in the carotid artery during 10 min with a TG emulsion (IL) added or not with heparin to stimulate LPL activity. As expected, by using isolated carotid infusions, there was no change in plasma TG or FA levels that might explain an effect of TG-enriched particles on food intake through indirect peripheral mechanisms. It has been indeed reported that oral ingestion of TG in rats decreased food intake through hepatic fatty acid oxidation [28] with modulation of the discharge rate of vagal afferents in rodents [29], [30]. By specifically targeting the forebrain, we demonstrate here that a short term infusion of TG emulsion lowered spontaneous food intake compared to controls (both S and S_H_), but only when heparin was added to the emulsion. This result strongly suggests a key role for FAs in the regulation of feeding. In regard to physiological relevance, it must be pointed out that fat ingestion is a relatively slow process compared to carbohydrates. Post-prandial TGs, from both exogenous and endogenous origins, start to significantly increase in bloodstream at least 40 to 50 min after a meal [23], [27]. It has been shown that fat alters meal patterns consistently with induction of short-term satiety signals [31]. Finally, because of the low amount of IL_H_ that we infused, cumulative food intake was transiently decreased during the first 5 h following infusion- but was similar in both IL_H_ and S_H_ groups at time 24 h post-infusion.

Additional experiments were performed to identify molecular mechanism underlying the effects of carotid IL_H_ infusions on food intake. We did not observe any increase in VMH FA concentrations during microdialysis or in FA composition by GS-MS in IL_H_ vs S_H_ rats. However, because of the very low concentrations of TG infused (20 µl/min, IL 20%) vs. the long periods of dialysate collections (30 min) it is likely that most or all of the FA crossing the blood-brain barrier would not significantly change extracellular FA levels but would, instead, be taken up by astrocytes which are the major site of FA oxidation in the brain [32]. Thus, it is not surprising to see no differences between IL_H_ and S_H_. On the other hand, small changes in FA concentrations could act as signaling molecules on VMH FA sensing neurons to alter their activity [14]. As we can see on figure 3, some hypothalamic nuclei involved in food intake regulation were activated after 10 min IL_H_ infusion in the carotid artery [1]. We can postulate that a local FA increase induces neuron activation in these areas *via* neurotransmitters release in ARC, PVN and VMN. This could explain the food intake decrease observed in ILH rats in the absence of neuropeptide mRNA changes in hypothalamus.

In order to identify potential molecular mechanisms underlying the FA effects on feeding, we performed additional experiments targeting CD36 acting as a long chain FA receptor [33], acylCoA synthesis or β-oxidation. VMH CD36 expression was decreased by ∼36% using shRNA against CD36, and we used a pharmacological approach to assess the role of acylCoA synthesis and β-oxidation, by co-infusion of triacsin C or etomoxir with IL_H_. Both CD36 knockdown and acylCoA synthesis inhibition prevented the feeding inhibition of intracarotid IL_H_, while inhibition of long chain FA transport into mitochondria with reduction of β-oxidation did not reverse the IL_H_ inhibition of feeding, at 5 h after refeeding.

A role for CD36 as a key actor of FA sensing in hypothalamus have been previously reported *in vitro* [14]. At least 50% of the FA sensing in VMH neurons was attributable to the interaction of long chain FA with CD36, while only ∼20% was attributable to intracellular metabolism of FA, including the inhibition of long chain FA acylCoA synthase (ACS) with triacsin C [14]. However, other FA transporters are also expressed in hypothalamus [34]. Since inhibition of CD36 reduces neuronal FA sensing by at least 50%, but inhibition of the first step of intracellular FA metabolism (acylCoA synthase) produces less of 20% inhibition of FA sensing [14], it is likely that CD36 functions as a receptor rather than transporter. This function has been documented in taste buds [35], [36]. The fact that inhibition of acylCoA synthase had an equivalent effect on IL_H_-induced inhibition of feeding as did reduction in VMH CD36 but has a much smaller effect on neuronal FA sensing suggests that this inhibition may be acting on both astrocytes and neurons. Since astrocytes can produce ketone bodies [37]which might then affect neuronal FA sensing, it is possible that some of the effects of IL_H_ infusions might be mediated by FA-induced ketone production by astrocytes. In neurons, an increase in acylCoA synthase activity might regulate neuronal activity through modulation of ionic channels.For example oleylCoAis a modulator of K_ATP_ channels [38] and oleic acid inhibits or activates hypothalamic FA sensing neurons through modulation of chloride [39] or K_ATP_ channels [40], respectively.

Finally, we found that inhibition of CPT1 activity and thus β-oxidation by etomoxir had a transient effect on food intake: there was a reversal of Intralipid-induced feeding inhibition at 1 h (similarly to shRNA against CD36 and triacsin C data), but not at 5 h.Taking together, as with neuronal FA sensing [14], inhibition of this critical step in FA oxidation does not appear to be required for the regulation of feeding by FA. The blockade of ILH effect on food inhibition by etomoxir, at 1 h after refeeding, is in contradiction with Obici et al [41] who found that inhibition of hypothalamic CPT1 activity substantially reduced food intake [41]. It is difficult to compare our studies with theirs given a variety of methodological differences. However, our method of intracarotid infusion of FA mimics the way in which this substrate is normally delivered to the brain. Also, since we did not measure VMH FA oxidation directly, there is no way to know the degree to which our infusions of etomoxir actually inhibited such oxidation.

**Figure 6:**
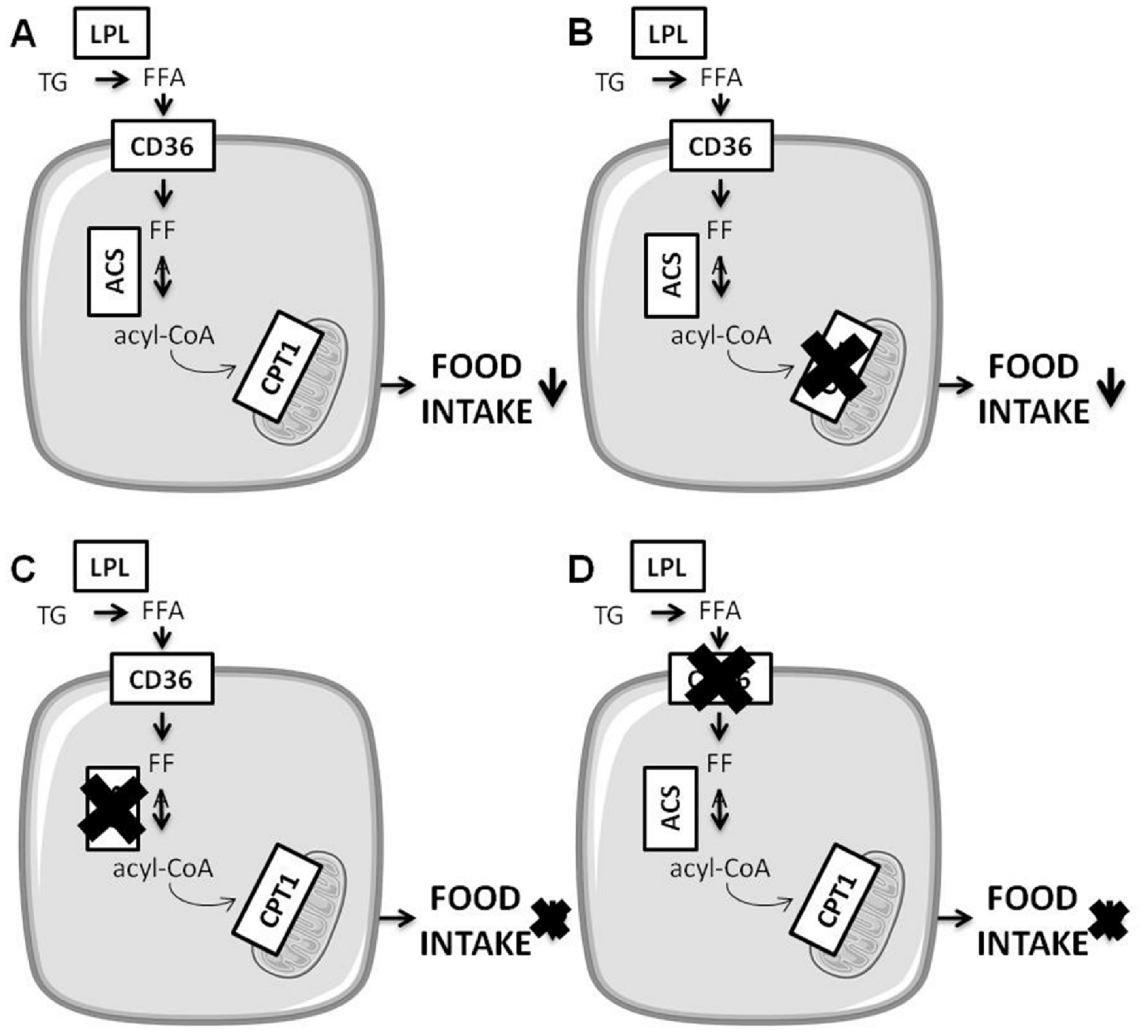
Schematic representations of proteins mediating FA effect on food intake in a fatty acid sensing cell.

In conclusion our data demonstrate that short term IL_H_ infusion via the carotid artery lowers food intake during refeeding in rats. This may mimic what happens in post-prandial state when TG-enriched lipoproteins increase in the bloostream with localhydrolysis by hypothalamic LPL providing FA to FA sensing neurons. We also found that both CD36 and ACS played regulatory roles in the inhibitory effects of intracarotid IL_H_ on food intake, whereas β-oxidation was not required (Figure 6). Finally, our data reinforce the role of CD36 as a major effector of hypothalamic FA sensing both *in vitro* and *in vivo* and as a potential target for the central regulation of food intake.
